# Country-level interventions for the prevention and management of hypertension through the modification of social determinants of health: a systematic review protocol

**DOI:** 10.1186/s13643-020-01392-9

**Published:** 2020-06-24

**Authors:** Fateme Arabi Basharic, Ali Janati, Mohammad Zakaria Pezeshki, Rahim Khodayari-Zarnaq, Fatemeh Sadeghi-Ghyassi, Masoumeh Gholizadeh

**Affiliations:** 1 Iranian Center of Excellence in Health Management (IceHM), Department of Health Services Management, School of Management and Medical Informatics, Tabriz University of Medical Sciences, Tabriz, Iran; 2 Social Determinants of Health Research Center, Health Management and Safety Promotion Research Institute and Department of Community & Family Medicine, Tabriz Medical School, Tabriz University of Medical Sciences, Tabriz, Iran; 3 Tabriz Health Services Management Research Center, Health Management and Safety Promotion Research Institute, Tabriz University of Medical Sciences, Tabriz, Iran; 4 Research Center for Evidence Based Medicine, Tabriz University of Medical Sciences, Tabriz, Iran

**Keywords:** Social determinants of health, Policy, Hypertension

## Abstract

**Background:**

Hypertension is one of the public health challenges. Various risk factors are associated with hypertension, including social demographics, geographical location, health behaviours, and social stress. Interventions in the social determinants of health can improve hypertension and health promotion. Accordingly, different sectors such as agriculture, housing, education, and transportation should cooperate. This systematic review examines policies as a set of activities and actions/interventions aimed at the modification of the social determinants of health to prevent hypertension.

**Methods:**

A systematic search will be conducted in Medline (via Ovid), PubMed, EMBASE, Cochrane Library, ProQuest Dissertations & Theses, and scientific Persian databases including SID and Magiran. There will be no time restriction. The quality of selected studies will be assessed using an appropriate Joanna Briggs Institute (JBI) Critical Appraisal Checklists according to the type of studies. Two independent researchers will carry out screening and quality assessment. Disagreement between two researchers will be resolved by a third party.

**Discussion:**

Recommendations will be made for policymakers in order to make better evidence-based decisions about the prevention and management of hypertension with regard to the social determinants of health.

**Systematic review registration:**

PROSPERO CRD42020152298

## Background

The non-communicable diseases are one of the most important health and development challenges in the twenty-first century, both affecting the individuals, and social and economic structure of countries, especially low- and middle-income countries [1]. Among the non-communicable diseases, hypertension is one of the public health challenges. The major factors contributing to the development of hypertension and its complications are social determinants of health such as globalisation, urbanisation, ageing, income, and housing causing behavioural risk factors. On the other hand, behavioural risk factors such as unhealthy diet, smoking, physical inactivity, and harmful alcohol consumption cause obesity and hypertension, which finally lead to cardiovascular diseases (heart attacks, strokes, and heart failure) [2].

The social determinants of health are “conditions in which the individuals are born, grow, live, work, and grow old” [3, 4]. A range of social, ecological, commercial, political, and cultural factors affecting the health are identified as determinants of health [5]. These conditions are often distributed in a disparate way among the individuals and social groups, resulting unequal outcomes of health [6]. These are complex and interactive factors that contribute to the current state of health, the chance of health maintenance and causing diseases. Factors such as housing, environmental state, genetics, income, education, and relationships with friends and family have a remarkable effect on the health [5]. The effect of social determinants on the health is far greater than the performance of the health system. Investing in providing these services in social determinants widely improves the community health indices [7]. Studies show that investing in social determinants of health such as housing and income support will provide positive results [4, 8]. The social determinants of health are established by political systems, resources, and value systems. The multi-level interventions in social determinants of health in the form of policies and plans can improve the outcomes related to the behaviour, risk, damage, well-being, quality of life, and health equity [9].

The College of American Physicians suggests policymakers to adopt a “Health in All Policies” approach and support the integration of health considerations into the community planning decisions via the use of “health impact assessment” [4]. Accordingly, different sectors (such as trade, agriculture, housing, education, transportation, and environment) should cooperate to adopt policies in order to protect the individuals and minimise their exposure to risks. For example, the use of fiscal policies to reduce tobacco use, environmental and labour laws to reduce exposure to smoke is developed and none of these activities is in the main range of health system [10]. On April 7, 2013, the World Health Day, World Health Organisation (WHO) launched a campaign to manage and prevent hypertension, which encouraged policymakers and other stakeholders to prioritise prevention, early diagnosis, and hypertension management in national policies, plans, and activities [11].

Studies show that various risk factors are associated with hypertension, including social demographics (higher age, gender, low education, and family income) [12–17], geographical location (urban accommodation) [12, 18], body weight status, health behaviours, and psychological and social stress and support [12]. Some studies show that hypertension is associated with psychological stress such as depression [12, 19], low level of satisfaction with life [12, 20, 21], low social cohesion [12], and lack of social support [12, 22]. The interaction of social conditions, individual lifestyles, and biological factors is critical in health policy [23].

In order to reduce the negative health outcomes associated with social determinants of health, a comprehensive approach is needed including support of public policies, better understanding by physicians, interpersonal communications improvement, organisational and social participation, appropriate financing, health considerations in the community planning and development, and robust planning in research and real evidence collection. The specific objectives of this review are as follows:

1. To identify successful interventions and policies aimed at modifying social determinants of health in the various social contexts and countries around the world that reduce the incidence, prevalence, and severity of hypertension

2. To document the reasons for the success of these policies and interventions

## Methods/design

### Study method

The systematic review protocol has been developed according to the Preferred Reporting Items for Systematic Reviews and Meta-Analyses Protocols (PRISMA-P) guidelines [24] (see Additional file 1). This protocol has been registered in PROSPERO (registration number CRD42020152298).

### Research questions

1. What policies and interventions have been implemented aimed at controlling the social determinants of health in various countries around the world as measures to reduce the incidence of hypertension?
2. What has led to the success of various interventions within one country or in different countries around the world?

### Eligibility criteria

We will include studies that focused on the policies applied to intervene in the social determinants of health for the prevention and management of hypertension. The policies are specific decisions, plans, and objectives pursued by governments, organisations, and institutions for the prevention and management of hypertension. This systematic review examines policies as a set of activities and actions/interventions aimed at the modification of the social determinants of health to prevent hypertension.

The criteria for study eligibility in this review including population, context, and outcome are described below.

### Population

All countries that have implemented policies/interventions in their population to control or modify social determinants of health as a measure against hypertension.

### Intervention

The policies and interventions that have been implemented in different settings including communities, worksites, schools, and health care organisations. These policies/interventions will include economic, social, and nutrioktional policies; educational programmes; and other policies that are effective for the prevention and management of hypertension. This study examines policies and interventions in the ten social determinants of health. These ten social determinants of health will include food, addiction, transportation, social support, stress, social exclusion, early life, work, social gradient, and unemployment.

### Context

The study’s context is all countries in the world with the rising prevalence of hypertension.

### Outcome

The outcomes are all policies and interventions that have succeeded in reducing the incidence/prevalence of hypertension or average blood pressure of various age groups over time through modifications of social determinants of health, and the documented reasons for each successful policy/intervention.

### Types of studies

All original research papers (quantitative, qualitative, and mixed method) aimed to intervene in the social determinants of health for the prevention and management of hypertension will be included. Studies that are focused on other non-communicable diseases will be excluded. Studies with inadequate or incomplete data will be excluded from the study. English and Persian language studies will also be included.

### Search strategy

The following databases will be searched: Medline (via Ovid), PubMed, EMBASE, Cochrane Library, and ProQuest Dissertations & Theses. Persian database including SID and Magiran will be searched. For each database, the appropriate search strategy will be implemented. The search strategy will include both free text and controlled vocabularies. We will hand-search reference lists of included studies and reference lists of relevant systematic reviews. We will supplement the electronic database searches with website and grey literature searches. We will search websites of relevant institutions and organisations such as the World Health Organisation. Other websites might be reviewed when undertaking the research. No date of publication restriction will be applied. An initial search strategy of Medline (Ovid) is given as additional file 2.

### Study selection

All retrieved papers will be imported in the Endnote software. After eliminating the duplicated papers, the titles and abstracts of the papers will be screened according to the inclusion and exclusion criteria and eligible studies will be selected. The selected papers will be reviewed in full text, and the final decision will be made regarding the study inclusion and exclusion criteria. The retrieved papers will be screened by two researchers (F-A-B and M-GH). Any disagreement between the two researchers will be resolved by a third party (A-J). The studies that met the inclusion criteria will be assessed for their quality, and the data of included studies will be extracted in accordance with the data extraction form. The studies that are not eligible for inclusion in the study will be reported separately in the table with reasons.

### Quality assessment

The quality of the included studies will be assessed by an appropriate Joanna Briggs Institute (JBI) Critical Appraisal Checklists according to the type of studies [25]. The JBI has separate checklists for cohort studies (11 criteria), cross-sectional studies (8 criteria), case-control studies (10 criteria), RCT studies (13 criteria), quasi-experimental studies (9 criteria), and qualitative studies (10 criteria). Options for checklist responses included “yes”, “no”, “unclear”, or “not applicable”. For example, the following items will be used to appraise cohort and cross-sectional studies: appropriateness of inclusion criteria, description of participants and setting, valid and reliable measurement of exposure, objective and standard criteria used, identification of confounder, strategies to handle confounder, outcome measurement, and appropriate statistical analysis. The risk of bias for study will be independently assessed by two reviewers (F-A-B and R-KH-Z). Any disagreement between the two authors will be resolved by the third party (A-J). According to the quality, studies will be reported in separate tables. The quality of studies will be graded high, medium, low:

High—a rigorous and robust scientific approach

Medium—some flaws and medium scientific value

Low—serious flaws and poor scientific value

### Data extraction

A data extraction form will be designed for data collection. The extracted data will be collected by using Excel 2016.The extracted data from studies will include items such as the authors’ name, publication year, country, context, study design, study methodology, target group, number of participants, policies, and actions taken and implemented. These items will be selected according to the key questions of the study. The data extraction will be done by all of the authors (F-A-B, M-GH, A-J, MZ-P, MZ-P, and F-S-GH). The draft of data extraction form will be modified during the data extraction process by the team.

### Data synthesis

Using information taken by the data extraction table, we will conduct a thematic analysis. Thematic analysis is appropriate for this review for two reasons. It fits our current objective of aggregating existing evidence, and also, thematic analysis has good transparency in delivering accessible results. The thematic analysis involves three steps of analysis. First, the data are classified. Then, the similarities between the data are identified. The third stage of thematic analysis includes the development of analytical themes. The data extracted (including all successful actions, initiatives, programmes in social determinants of health and successful reasons for each outcome) are classified. Then, the similarities between those are identified. In the final will be determined themes.

## Discussion

Hypertension is the most important preventable cause of cardiovascular disease in the world [26]. In Iran, as a developing country, cardiovascular disease is the leading cause of death [27, 28]. It is necessary to use the social determinants of health approach, to identify policies that prevent incidence of hypertension. We expect that the results of this systematic review produce valuable evidence for policymakers in health policy. These policies will be useful for policymakers, officials and planners, which are in cooperation and consultation with citizens and organisations and government, to suggest policies for the prevention of hypertension and reduce the burden of disease imposed on society.

## Strengths and limitations of this study

The strengths of this review include a systematic approach to search for articles. The quality of studies is assessed using appropriate and valid tools. This review provides a credible documentary for policymakers to make evidence-based decisions. The limitation of this review is that it includes studies published in both English and Persian, and some countries publish articles in their official language, so valuable policies may not be included in this review and may be lost.

## Supplementary information

**Additional file 1:.** PRISMA-P-checklist

**Additional file 2:.** Search strategy

## Data Availability

Not applicable

## Abbreviations

JBI: Joanna Briggs Institute
PRISMA: Preferred Reporting Items for Systematic Review and Meta-Analysis Statement
PROSPERO: International Prospective Register of Systematic Reviews
SID: Scientific Information Database
