# Supplementary material for: Country-level interventions for the prevention and management of hypertension through the modification of social determinants of health: a systematic review protocol
Source: Syst Rev. 2020 Jun 24;9:152. doi: 10.1186/s13643-020-01392-9 (PMC7315529; doi:10.1186/s13643-020-01392-9)
Supplement: Supplementary file 2 — Additional file 2:. Search strategy [file 13643_2020_1392_MOESM2_ESM.docx]

**Additional file 2**

Search strategy

Medline (OVID)

1 exp "Social Determinants of Health"

2 Social Determinant$.tw.

3 1 or 2

4 exp Unemployment

5 (Unemploy$ or Underemploy$).tw.

6 4 or 5

7 exp Employment/

8 (employ$ or work$ or job$ or career$ or Occupation$).tw.

9 exp Work/

10 7 or 8 or 9

11 social gradient$.tw.

12 stress.tw.

13 social exclusion$.tw.

14 social support$.tw.

15 exp Social Support/

16 14 or 15

17 exp Substance-Related Disorders/

18 (addiction$ or abuse$).tw.

19 17 or 18

20 exp Food/

21 Food$.tw.

22 20 or 21

23 transport$.tw.

24 early life$.tw.

25 early child$.tw.

26 24 or 25

27 3 or 6 or 10 or 11 or 12 or 13 or 16 or 19 or 22 or 23 or 26

28 exp Hypertension/

29 (hypertension$ or High Blood Pressure$).tw.

30 28 or 29

31 exp Policy/

32 Polic$.tw.

33 31 or 32

34 27 and 30 and 33
